# Benzimidazole derivative small-molecule 991 enhances AMPK activity and glucose uptake induced by AICAR or contraction in skeletal muscle

**DOI:** 10.1152/ajpendo.00237.2016

**Published:** 2016-08-30

**Authors:** Laurent Bultot, Thomas E. Jensen, Yu-Chiang Lai, Agnete L. B. Madsen, Caterina Collodet, Samanta Kviklyte, Maria Deak, Arash Yavari, Marc Foretz, Sahar Ghaffari, Mohamed Bellahcene, Houman Ashrafian, Mark H. Rider, Erik A. Richter, Kei Sakamoto

**Affiliations:** ^1^Nestlé Institute of Health Sciences SA, EPFL Innovation Park, Lausanne, Switzerland;; ^2^Université catholique de Louvain and de Duve Institute, Brussels, Belgium;; ^3^Department of Nutrition, Exercise and Sports, Faculty of Science, University of Copenhagen, Copenhagen, Denmark;; ^4^School of Life Sciences, Ecole Polytechnique Fédérale de Lausanne, Lausanne, Switzerland;; ^5^Experimental Therapeutics and Division of Cardiovascular Medicine, Radcliffe Department of Medicine, University of Oxford, Oxford, United Kingdom;; ^6^U1016, Institut National de la Santé et de la Recherche Médicale, Institut Cochin, Paris, France;; ^7^UMR8104, Centre National de la Recherche Scientifique, Paris, France; and; ^8^Université Paris Descartes, Paris, France

**Keywords:** AMP-activated protein kinase, LKB1, compound 13, A769662, 991, ex229, 5-aminoimidazole-4-carboxamide riboside

## Abstract

AMP-activated protein kinase (AMPK) plays diverse roles and coordinates complex metabolic pathways for maintenance of energy homeostasis. This could be explained by the fact that AMPK exists as multiple heterotrimer complexes comprising a catalytic α-subunit (α1 and α2) and regulatory β (β1 and β2)- and γ (γ1, γ2, γ3)-subunits, which are uniquely distributed across different cell types. There has been keen interest in developing specific and isoform-selective AMPK-activating drugs for therapeutic use and also as research tools. Moreover, establishing ways of enhancing cellular AMPK activity would be beneficial for both purposes. Here, we investigated if a recently described potent AMPK activator called 991, in combination with the commonly used activator 5-aminoimidazole-4-carboxamide riboside or contraction, further enhances AMPK activity and glucose transport in mouse skeletal muscle ex vivo. Given that the γ3-subunit is exclusively expressed in skeletal muscle and has been implicated in contraction-induced glucose transport, we measured the activity of AMPKγ3 as well as ubiquitously expressed γ1-containing complexes. We initially validated the specificity of the antibodies for the assessment of isoform-specific AMPK activity using AMPK-deficient mouse models. We observed that a low dose of 991 (5 μM) stimulated a modest or negligible activity of both γ1- and γ3-containing AMPK complexes. Strikingly, dual treatment with 991 and 5-aminoimidazole-4-carboxamide riboside or 991 and contraction profoundly enhanced AMPKγ1/γ3 complex activation and glucose transport compared with any of the single treatments. The study demonstrates the utility of a dual activator approach to achieve a greater activation of AMPK and downstream physiological responses in various cell types, including skeletal muscle.

amp-activated protein kinase (AMPK) is an important energy sensor that maintains energy homeostasis by coordinating metabolic pathways/processes in response to energy supply and demand (38). During metabolic stress, the cellular AMP-to-ATP ratio increases, leading to the activation of AMPK, which, in turn, switches off energy-consuming anabolic pathways and switches on catabolic pathways to restore ATP levels. One of the most physiological metabolic stresses that activates AMPK is skeletal muscle contraction, which contributes at least partly to an increase in glucose uptake by promoting glucose transporter (GLUT)4 translocation to the plasma membrane by phosphorylating candidate mediators such as TBC1 domain family member 1 and FYVE domain-containing phosphatidylinositol 3-phosphate 5-kinase (15, 21, 25, 30, 32).

AMPK is a heterotrimer comprising a catalytic α-subunit and regulatory β- and γ-subunits. Multiple isoforms encoded by distinct genes exist (α1 and α2, β1 and β2, and γ1, γ2, and γ3), which can theoretically form several distinct heterotrimeric combinations (14, 38). Isoform expression varies among cells/tissues and also species (40, 45), with α1, β1, and γ1 appearing in general to be ubiquitously expressed isoforms. Some isoforms are expressed in a cell/tissue-specific/restricted manner. For example, α2 is more predominantly expressed in skeletal muscle (26) and γ3 is exclusively detected in skeletal muscle, especially in glycolytic muscle fibers (5, 49). The γ-subunits contain four tandem cystathionine β-synthase (CBS) repeats that bind adenine nucleotides. AMPK activity increases upon phosphorylation of a conserved threonine residue in the T-loop (T172) (14). Binding of ADP and/or AMP to CBS repeats induces conformational changes that promote net T172 phosphorylation by the enhancement of T172 phosphorylation and suppression of T172 dephosphorylation (13, 27, 47). Moreover, the binding of AMP (but not ADP) further increases AMPK activity by direct allosteric stimulation (13). The major upstream kinase phosphorylating T172 in metabolic tissues (e.g., muscle and liver) is a complex containing LKB1 (33, 37), which appears to be constitutively active (1, 31).

AMPK activation has favorable effects on carbohydrate and lipid metabolism in the skeletal muscle and liver, and there has been keen interest in developing AMPK-activating drugs for therapeutic use in the treatment of metabolic diseases, such as type 2 diabetes. Cool et al. (6) described the identification of the thienopyridone A769662, the first small-molecule direct activator of AMPK. A769662, like AMP, inhibits T172 dephosphorylation (12, 34), although it does not bind to adenine nucleotide-binding sites on the γ-subunit. The oral absorption of this compound is poor. However, when injected intraperitoneally, it displayed antidiabetic effects, including reduced weight gain, decreased plasma glucose and triglycerides, and decreased liver triglycerides in obese mice (6). In addition, A769662 is a valuable research tool, although its utility is limited by the marked selectivity toward complexes containing β1 rather than β2 isoforms (36). Consequently, it robustly stimulates AMPK in mouse hepatocytes (where β1 is predominantly expressed) and modulates lipid metabolism via an AMPK-dependent mechanism (9, 18). In mouse skeletal muscle, β2 is predominantly expressed, whereas only a modest amount of β1 is detectable (39, 43). It has been shown that incubation of isolated mouse skeletal muscle ex vivo with A769662 failed to stimulate glucose uptake at 100–200 μM. At a much higher concentration (2 mM), A769662 stimulated glucose uptake; however, it was mediated through an AMPK-independent/phosphoinositide 3-kinase-dependent mechanism due to unknown off-target effect(s) (43). Another small-molecule AMPK activator, 991 [also known as ex229 (22)], has recently been described. Structural studies have shown that 991 binds to a site formed between the small lobe of the α-subunit kinase domain and the β-subunit carbohydrate-binding module (4, 46), which is also the binding site for A769662, termed the allosteric drug and metabolite-binding site (ADaM) (23). 991 is 5- to 10-fold more potent than A769662 in cell-free assays (46) and has been shown to stimulate AMPK activity of both β1- and β2-containing complexes and to increase glucose transport in isolated mouse skeletal muscle ex vivo (22).

Establishing a means to augment cellular AMPK activation would potentially enhance the therapeutic effects of future AMPK activators and in fundamental research allow more robust biochemical and functional analyses in intact cells/tissues. We (8, 11, 41) and others (35) have shown that dual AMPK-activating compound treatment [A769662 and 5-aminoimidazole-4-carboxamide riboside (AICAR)] resulted in additive/synergistic increases in cellular AMPK activity and downstream physiological effects, such as inhibition of lipogenesis in hepatocytes (8). One limitation of the A769662 and AICAR dual treatment is that the additive or synergistic effects can only be produced in cells where β1 is predominantly expressed (e.g., mouse hepatocytes), as the effects were absent in β1-deficient cells (8). In the present study, we explored whether treatment with the potent ADaM site-binding agent 991 would enhance the effect of the canonical adenine nucleotide binding-mediated activation of AMPK by AICAR and contraction in mouse skeletal muscle. Given that activation of γ3-containing complexes (especially α2β2γ3) is associated with AICAR- and contraction-mediated glucose uptake (2, 3), we assessed the effect of 991 alone and in combination with AICAR or contraction on activating AMPKγ1 and AMPKγ3 complexes. We demonstrated that a low dose of 991 results in only a modest or negligible activation of γ1- and γ3-containing AMPK complexes in mouse skeletal muscle. However, strikingly, dual treatment with 991 and AICAR or 991 and contraction profoundly enhanced AMPK activation and glucose transport compared with single agonist treatments.

## MATERIALS AND METHODS

### 

#### Materials.

AICAR was from Toronto Research Chemicals. A769662 was from Selleck Chemicals. Compound 13 was obtained as previously described (18). 991 (5-{[6-chloro-5-(1-methylindol-5-yl)-1H-benzimidazol-2-yl]oxy}-2-methyl-benzoic acid; CAS no. 129739-36-2) as obtained as previously described (7). Protein G Sepharose was from GE Healthcare, and FLAG-M2 resin from Sigma-Aldrich. ECL reagent and P81 filter papers were obtained from GE Healthcare. [γ-^32^P]-ATP was from Perkin-Elmer. AMARA, LKBtide, and Sakamototide substrate peptides were synthesised by GL Biochem. The COS1 cell line was obtained from American Type Culture Collection, and the C_2_C_12_ cell line was obtained from Sigma-Aldrich. Frozen tissues or extracts from AMPKα1-/α2-, AMPKβ1-/β2-, and AMPKγ3-deficient mice were obtained from Benoit Viollet and Marc Foretz (Institut National de la Santé et de la Recherche Médicale, Institut Cochin, Paris, France), Gregory Steinberg (McMaster University, Hamilton, ON, Canada), and Alexander Chibalin and Juleen Zierath (Karolinska Institutet, Stockholm, Sweden), respectively. All cell culture reagents were purchased from Thermo Fisher Scientific, and all other chemicals were from Sigma-Aldrich unless otherwise stated.

#### Antibodies.

AMPKα1/α2 (no. 2532), phosphorylated (p)T172 AMPKα (no. 2535), AMPKβ1 (no. 4182), AMPKβ2 (no. 4148), AMPKβ1/β2 (no. 4150), pS79 acetyl-CoA carboxylase (ACC; no. 3661), ACC (no. 3676), pS792 regulatory associated protein of mechanistic target of rapamycin (RAPTOR; no. 2083), and RAPTOR (no. 2280) were from Cell Signaling Technology. FLAG (F7425) and α-tubulin (T6074) antibodies were from Sigma-Aldrich. AMPKγ2 (sc-19141) and creatine kinase (sc-15161) antibodies were from Santa Cruz Biotechnology. AMPKα1 (no. 07-350) and AMPKα2 (no. 07-363) antibodies were obtained from Millipore. For immunoprecipitation, the antibody for AMPKβ1 (A4856) was from Sigma-Aldrich and the antibody for AMPKβ2 (MAB3808) was from R&D Systems. AMPKα1 and AMPKα2 antibodies used for immunoprecipitation were as previously described (33). LKB1 and salt-inducible kinase (SIK)3 antibodies have also been previously described (31). The following polyclonal antibodies were generated by YenZym Antibodies by immunizing rabbits with the indicated peptides: a combination of human AMPKγ3 peptide [residues S45–S65: *CSSERIRGKRRAKALRWTRQKS; a terminal cysteine (*C) was added to the peptide sequence to allow peptide conjugation to carrier proteins], mouse AMPKγ3 peptide (residues S44–S64: *CSSERTCAIRGVKASRWTRQEA), and AMPKγ1 peptide (residues E7–S26 of mouse AMPKγ1: *CESSPALENEHFQETPESNNS). The produced antibodies were affinity purified using respective antigen-specific peptide columns by YenZym Antibodies. Horseradish peroxidase-conjugated secondary antibodies were from Jackson ImmunoResearch.

#### Kinase inhibition screening.

Kinase inhibition screening was performed by the International Centre for Kinase Profiling (University of Dundee) using [γ-^33^P]phosphotransferase assays (MRC Protein Phosphorylation and Ubiquitylation Unit, http://www.kinase-screen.mrc.ac.uk/).

#### Molecular cloning.

The coding region of human AMPKγ1 (NM_002733), AMPKγ2 (NM_016203.3), and AMPKγ3 (NM_017431) was amplified from muscle RNA (Agilent) using a Superscript III 1 step RT PCR kit (Invitrogen). The resulting PCR products were either ligated into intermediate vector (pSC Agilent) or digested with the relevant restriction enzymes and ligated directly into the mammalian expression vector (pCMV5 acc. AF239249), which was modified to incorporate a NH_2_-terminal FLAG tag. Sequences of all clones were verified in house at the Nestlé Institute of Health Sciences using the BigDye Terminator 3.1 kit and 3500XL Genetic analyzer (Applied Biosystems).

#### Recombinant muscle-specific AMPK trimeric complex.

Human AMPK α2β2γ3 was expressed in *Esherichia coli* using a tricistronic construct activated with Ca^2+^/calmodulin-dependent protein kinase kinase-β in vitro and then purified and assayed as previously described (18).

#### Animals.

Animal experiments were approved by the local ethics committee and conducted in accordance with the European Convention for the Protection of Vertebrate Animals used for Experimental and Other Scientific Purposes. Protocols used were approved by the Service Vétérinaire Cantonal (Lausanne, Switzerland) under license VD2841 or approved by the University of Oxford Animal Care and Ethical Review Committee and conformed with the United Kingdom Animals Scientific Procedures Act 1986, incorporating European Directive 2010/63/EU (no. 30/2977), or approved by the Danish Animal Experiments Inspectorate or approved by the University of Paris-Descartes ethics committee (no. CEEA34.BV.157.12) and performed under French authorization to experiment on vertebrates (no. 75-886) in accordance with European guidelines. The generation of AMPKγ1^−/−^ mice has been previously described (10). Global AMPKγ2^−/−^ mice were generated by deleting the entire exon 7 of the gene encoding AMPKγ2 in R299Q knockin mice using Sox2cre-driven excision (48). Mice were maintained on a standard chow diet and 12:12-h light-dark cycle.

#### Cell and tissue extract preparation.

After treatment, cells were washed once with PBS and scraped into ice-cold lysis buffer [containing 50 mM Tris·HCl (pH 7.5), 1 mM EDTA, 1 mM EGTA, 270 mM sucrose, 1% (wt/vol) Triton X-100, 20 mM glycerol-2-phosphate, 50 mM NaF, 5 mM Na_4_P_2_O_7_, 1 mM DTT, 0.1 mM PMSF, 1 mM benzamidine Cl, 1 μg/ml microcystin-LR, 2 μg/ml leupeptin, and 2 μg/ml pepstatin A]. Frozen tissues were homogenised using a polytron PT 2500 E (Kinematica) in ice-cold lysis buffer. Cell/tissue lysates were clarified by centrifugation at 3,500 *g* for 15 min at 4°C, and protein concentration was measured using Bradford reagent and BSA as a standard.

#### Cell culture.

COS1, mouse embryonic fibroblast, and C_2_C_12_ cells were maintained in DMEM GlutaMAX (Thermo Fisher Scientific) supplemented with 10% (vol/vol) FBS and antibiotics. C_2_C_12_ myoblasts were differentiated into myotubes by 7 days of culture in DMEM GlutaMAX supplemented with 2% (vol/vol) horse serum, 100 U/ml penicillin G, and 100 μg/ml streptomycin. COS1 cells were grown in 6-cm dishes and transfected at 60–70% confluency with 3.7 μg plasmid prebound to 10.5 μg polyethylenimine in 50 mM HEPES (pH 7.4) and 150 mM NaCl. Cell culture medium was changed once at 24 h after transfection, and cells were left for an additional 24 h before compound treatment. Mouse embryonic fibroblast cells were grown in 10-cm dishes and treated as indicated at 80–90% confluency. Primary mouse hepatocytes were isolated from C57BL/6NTac male mice (Taconic) by collagenase perfusion, as previously described (18, 29). Hepatocytes were seeded in medium 199 containing 100 U/ml penicillin G, 100 μg/ml streptomycin, 0.1% (wt/vol) BSA, 10% (vol/vol) FBS, 10 nM insulin, 200 nM triiodothyronine, and 100 nM dexamethasone. Hepatocytes were left for attachment (3–4 h) and cultured overnight in medium 199 supplemented with antibiotics and 100 nM dexamethasone. Cells were used for experiments the following morning.

#### Immunoblot analysis.

Cell or tissue lysates were denatured in Laemmli buffer at 95°C for 5 min and separated by Tris-glycine SDS-PAGE and transferred onto polyvinylidene difluoride membranes. Membranes were blocked for 1 h at room temperature in 20 mM Tris·HCl (pH 7.6), 137 mM NaCl, and 0.1% (vol/vol) Tween 20 (Tris-buffered saline with Tween 20) containing 5% (wt/vol) skimmed milk. Membranes were incubated in primary antibody prepared in Tris-buffered saline with Tween 20 containing 5% (wt/vol) BSA or skimmed milk overnight at 4°C. Signal detection was performed using horseradish peroxidase-conjugated secondary antibodies and ECL.

#### Immunoprecipitation and kinase activity assay.

Cell or tissue lysates were incubated with the indicated antibodies (shown in Fig. 2F) precoupled to protein G-Sepharose for 1 h at 4°C. Immune complexes were pelleted at 500 *g* for 1 min and washed twice with 0.5 ml lysis buffer containing 500 mM NaCl and twice with 0.5 ml *buffer A* [50 mM HEPES (pH 7.4), 150 mM NaCl, 1 mM EGTA, and 1 mM DTT] and eluted with Laemmli buffer for analysis by immunoblotting or assayed directly for kinase activity. The AMPK activity assay was performed as previously described (19). Briefly, beads were incubated 45 min at 30°C under agitation in *buffer A* supplemented with 10 mM magnesium acetate, 100 μM ATP, and 200 μM AMARA peptide (NH_2_*-*AMARAASAAALARRR-COOH) in the presence of [γ-^32^P]ATP (1 μCi). Reactions were terminated by spotting reaction mixtures onto P81 filters and immersion in 75 mM phosphoric acid. Washed filters were dried, and ^32^P incorporation into the substrate AMARA peptide was measured by Cherenkov counting.

LKB1 and SIK3 assays were performed as previously described (31), and activity was measured using LKBtide and Sakamototide (16) as substrates, respectively.

#### Incubation, contraction, and glucose uptake in isolated mouse skeletal muscle.

Mice were euthanized by cervical dislocation, and extensor digitorum longus (EDL) muscles were rapidly removed and mounted on an incubation apparatus. The EDL muscle was incubated as previously described (20) in the presence of 991 or vehicle (0.1% DMSO) for 60 min with or without AICAR or in the presence of 991 or vehicle (0.1% DMSO) for 45 min with or without contraction evoked via electrical stimulation (1 Hz, 0.1-ms square wave 30-V pulses) (42) for 15 min. 2-Deoxy-[^3^H]glucose uptake was measured during the last 10 min of the incubation or contraction period of each experiment, as described previously (20).

#### Statistical analysis.

Results are expressed as means ± SE (unless otherwise stated) for the indicated number of individual experiments. Statistical significance was assessed using a paired two-sided Student's *t*-test (Fig. 3) or one-way ANOVA. When ANOVA revealed significant differences, further analysis was performed using Fisher's least-significant-difference post hoc test for multiple comparisons (Figs. 4–7). Differences between groups were considered statistically significant at *P* < 0.05.

## RESULTS

### 

#### Validation of AMPKα-, β-, and γ-isoform-specific antibodies for immunoblot analysis.

**Fig. 1.**
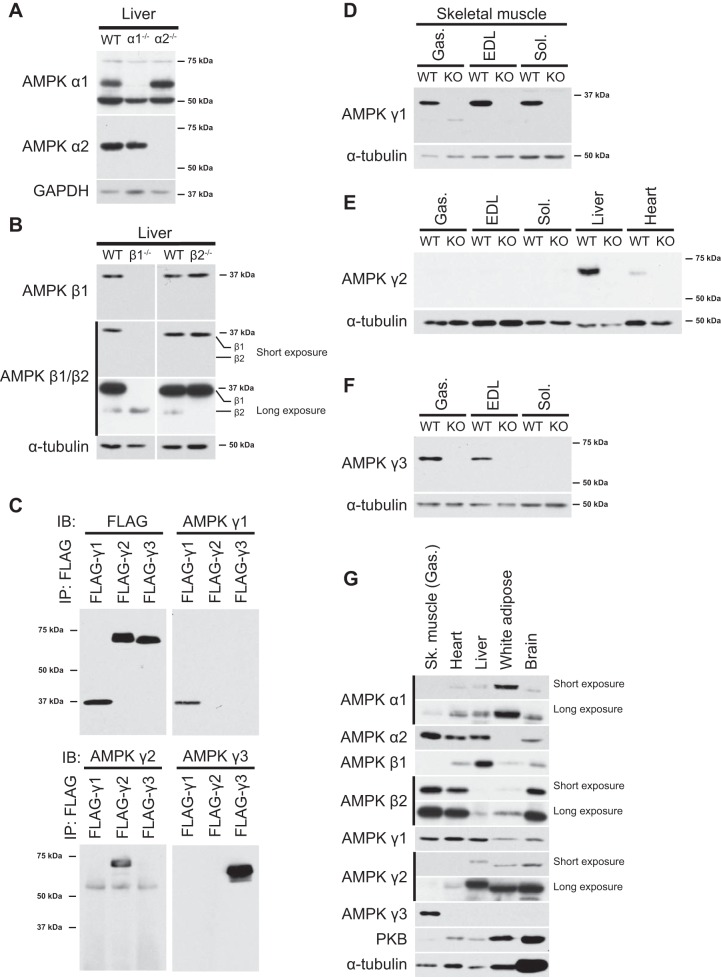
Validation of AMP-activated protein kinase (AMPK) isoform-specific antibodies for immunoblot (IB) analysis. *A* and *B*: IB analysis was performed with 20 μg liver tissue protein extracts from wild-type (WT) and AMPKα1/2 knockout (KO) mice (*A*) as well as AMPKβ1/β2 KO mice (*B*) using the indicated antibodies. IP, immunoprecipation. *C*: FLAG-tagged human AMPKγ1, AMPKγ2, and AMPKγ3 were ectopically expressed in COS1 cells, and cell extracts were generated. All three AMPKγ isoforms were immunoprecipitated with anti-FLAG antibody and immunoblotted with the indicated antibodies. *D–F*: IB analysis was performed with 20 μg of the indicated tissue extract proteins from WT and isoform-specific AMPKγ KO mice using the indicated antibodies. Gas, gastrocnemius; EDL, extensor digitorum longus; Sol, soleus. *G*: a panel of mouse tissue extracts (male C57BL/6NTac mice) was immunoblotted with the indicated antibodies. Sk, skeletal PKB, protein kinase B. Representative blots are shown from *n* = 1–3 mice/tissues.

To investigate the effect of dual treatments on AMPK isoform/complex-specific activity using cell or tissue extracts in subsequent experiments, we initially performed an extensive characterization/validation of the AMPK isoform-specific antibodies. We verified the specificity of AMPKα1, AMPKα2, AMPKβ1, and AMPKβ2 antibodies for immunoblot analysis using the respective isoform-specific AMPK knockout (KO) liver tissue extracts as controls (Fig. 1, *A* and *B*). The specificity of the AMPKγ antibodies has not been well documented, and we first assessed cross-reactivity of the antibodies using recombinant proteins. Individual constructs encoding FLAG-tagged human AMPKγ1, AMPKγ2, or AMPKγ3 were transfected in COS1 cells, and all three γ-isoforms were isolated from cell lysates using anti-FLAG antibody coupled to protein G-Sepharose. Immunoblot analysis of the FLAG-immunoprecipitates confirmed that similar amounts of each AMPK γ-isoform were isolated from the lysates (Fig. 1*C*, *top left*). We next immunoblotted the immunoprecipitates with AMPK γ1-, γ2-, or γ3-isoform-specific antibodies. As shown in Fig. 1*C* (*top right* and *bottom*), each antibody only detected the corresponding AMPK γ-isoform. We then tested the specificity of the γ antibodies against the endogenous subunit protein using tissue extracts from wild-type (WT) versus isoform-specific AMPKγ KO mice. As shown in Fig. 1, *D–F*, each isoform-specific antibody detected a band at the expected relative molecular mass in WT tissue extracts but not in the corresponding KO tissue extracts. AMPKγ1 was expressed similarly in the three muscle types measured: gastrocnemius (glycolytic/oxidative fibers mixed), EDL (predominantly containing glycolytic fibers), and soleus (predominantly containing oxidative fibers). AMPKγ3 was expressed in the gastrocnemius and EDL but not in soleus (Fig. 1*F*), consistent with previous reports (2, 49). Of note, AMPKγ2 was not detectable in skeletal muscle, although it could be detected in the liver and heart (Fig. 1*E*). Immunoblot analysis of a selection of mouse tissue extracts using the validated AMPK isoform-specific antibodies (Fig. 1, *A–F*) verified the exclusive expression of AMPKγ3 in skeletal muscle and predominant expression of AMPKα1 in white adipose (Fig. 1*G*). Predominant expression of AMPKβ1 in the liver and AMPKβ2 in the skeletal muscle, as well as the ubiquitous expression of AMPKγ1, was also confirmed. It has been previously reported that in mouse glycolytic skeletal muscle (EDL), β1 expression represents only ∼5% of total β-subunit expression (43), and β1 expression was not detectable with immunoblot analysis (20 μg lysates; Fig. 1*G*), even with longer film exposure (data not shown). AMPKγ2 was broadly expressed, but its expression appeared relatively low (considering the levels of loading control: α-tubulin and protein kinase B) in the heart and was not detectable in skeletal muscle (Fig. 1, *E* and *G*).

#### Validation of AMPK β- and γ-isoform-specific antibodies for immunoprecipitation.

**Fig. 2.**
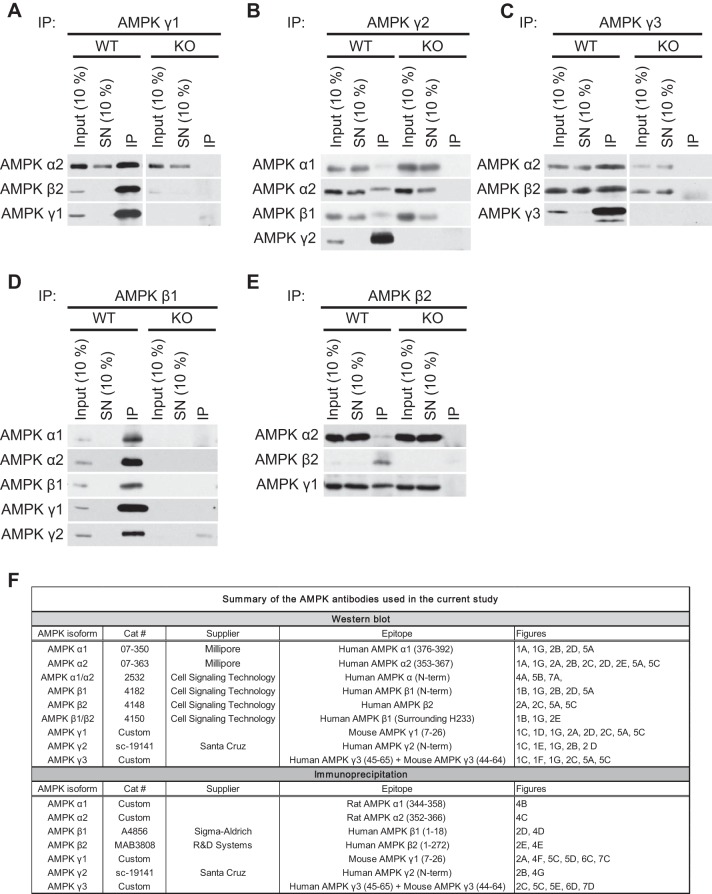
Validation of AMPK isoform-specific antibodies for IP. *A–E*: Endogenous AMPK complexes were immunoprecipitated using the indicated antibodies from 200 μg extract protein from the gastrocnemius muscle (*A* and *C*) or liver (*B*, *D*, and *E*) of the indicated AMPK WT or KO mice. IB analysis was performed on the input (10% = 20 μg), the supernatant (SN; 10% = 20 μg), and the immunoprecipitates (IP) with the indicated antibodies. *F*: summary table of the isoform-specific antibodies used in the present study.

We next examined the specificity and efficiency of the AMPK β- and γ-isoform-specific antibodies for immunoprecipitation using mouse tissue extracts [specificity of AMPKα1/α2 antibodies for immunoprecipitation has been previously described (33)]. We verified that each isoform-specific antibody immunoprecipitated the corresponding AMPK β- and γ-subunit proteins (using extracts from WT animals) in a specific and nearly complete manner (Fig. 2, *A–E*). There was no immunoreactive band/signal detected in extracts from the corresponding AMPK KO lysates and only a negligible amount of proteins detected in the supernatant that had been incubated with the corresponding antibody. Moreover, we confirmed that each AMPK β- and γ-isoform-specific antibody could precipitate its known interacting AMPK α- and β-subunit isoform(s) [i.e., AMPKα and AMPKβ proteins were identified in γ antibody immunoprecipitates (43); Fig. 2*A*] from the tissue extracts (Fig. 2, *A–E*).

In summary, we validated AMPK isoform-specific antibodies for both immunoblot analysis and immunoprecipitation. Given that we used several antibodies for different applications in the present study, a summary of the antibody list is shown in Fig. 2*F*.

#### Effect of a small-molecule AMPK activator, 991, on a panel of protein kinases in vitro.

**Fig. 3.**
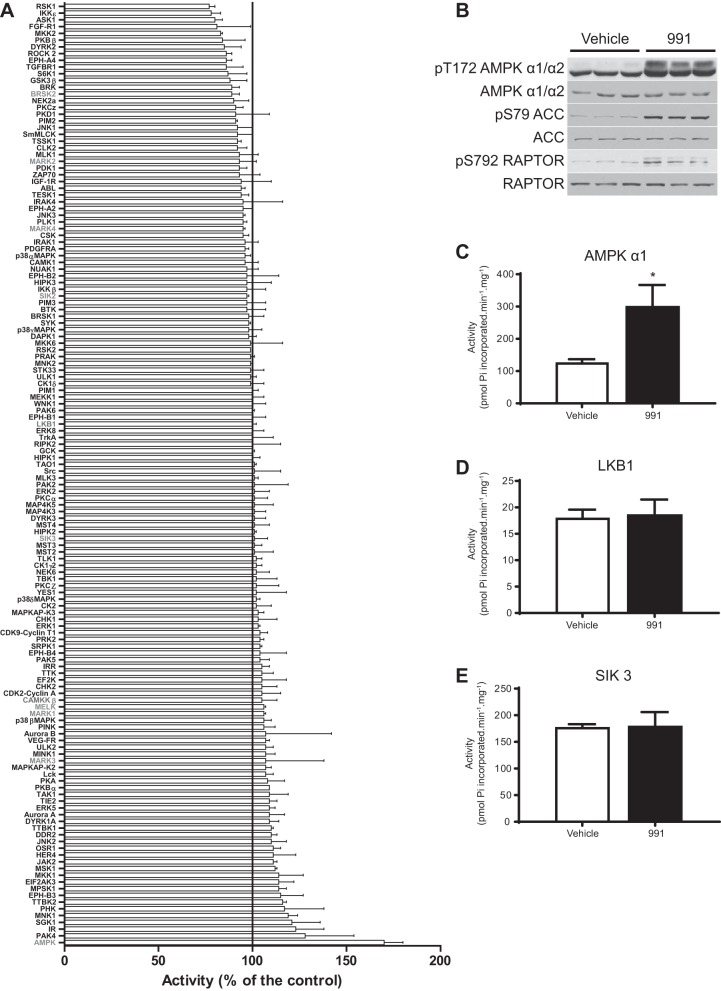
Effect of the small-molecule AMPK activator 991 on activity of a panel of 139 protein kinases. *A*: a kinase screen was performed by the International Centre for Kinase Profiling (University of Dundee). Assays were performed in the presence of 1 μM 991. Results are shown in rank order as mean percent activity compared with controls in the absence of compound ± SD. *B–E*: mouse embryonic fibroblasts were treated with vehicle (0.1% DMSO) or 991 (10 μM) for 1 h (3 independent plates for each condition). Cell lysates were immunoblotted with the indicated antibodies (*B*). The indicated kinases were immunoprecipitated from cell lysates, and in vitro kinase activity toward substrate peptide was measured (*C–E*) as described in materials and methods. ACC, acetyl-CoA carboxylase; RAPTOR, regulatory associated protein of mechanistic target of rapamycin; SIK3, salt-inducible kinase. Results are expressed as means ± SD. *P < 0.05.

We screened compound 991, a recently described potent AMPK activator (22, 46), at a concentration of 1 μM in cell-free assays against a panel of 139 protein kinases, including AMPK (Fig. 3*A*). As anticipated, recombinant AMPK complex [human α1(11–559)β2(1–272)γ1(11–331)] was activated ∼70% (highlighted in gray in Fig. 3*A*), whereas none of the other kinases, including upstream kinases for AMPK (LKB1 and Ca^2+^/calmodulin-dependent protein kinase kinase-β, highlighted in gray) and members of AMPK-related kinases (1) [BR serine/threonine kinase (BRSK)1, BRSK2, SIK2, SIK3, microtubule affinity regulating kinase (MARK)2, MARK3, MARK4, and maternal embryonic leucine zipper kinase, highlighted in gray] were significantly affected (Fig. 3*A*). We have confirmed that 991 robustly stimulates recombinant muscle-specific AMPKα2β2γ3 complex in a separate gold standard kinase activity assay in vitro [vehicle (DMSO): 496 ± 123 (SD) vs. 991, 3,380 ± 679 pmol P_i_ incorporated·min^−1^·mg^−1^, *P* < 0.05]. Furthermore, in mouse embryonic fibroblasts 991 increased the phosphorylation of AMPKα and its substrates ACC and RAPTOR (Fig. 3*B*). Consistent with this observation (Fig. 3*B*) and in vitro screen data (Fig. 3*A*), 991 robustly stimulated AMPKα1 activity (Fig. 3*C*) but not LKB1 or SIK3 (Fig. 3, *D* and *E*). These results suggest that 991 is a specific activator of AMPK at least in vitro and to a limited extent in intact cells.

#### Enhanced activation of cellular AMPK activity by dual compound treatment in hepatocytes.

**Fig. 4.**
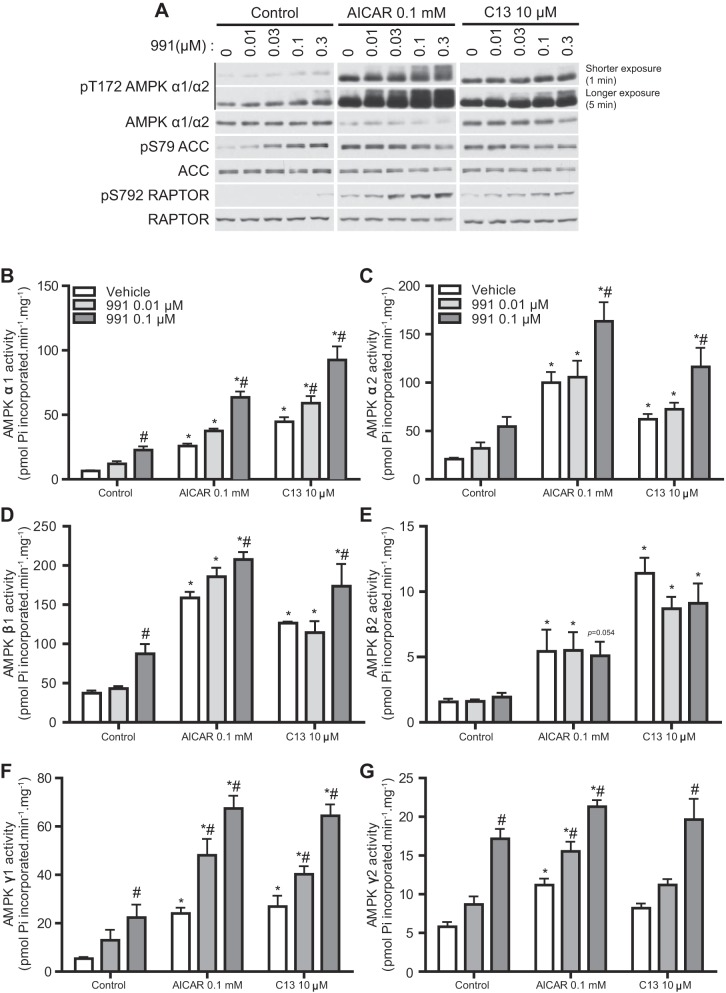
991 treatment enhances AMPK activity induced by 5-aminoimidazole-4-carboxamide riboside (AICAR) or C13 in hepatocytes. Hepatocytes were isolated from male C57BL/6NTac mice and cultured overnight as described in materials and methods. Hepatocytes were untreated (control) or treated with AICAR (0.1 mM) or C13 (10 μM) for 1 h in the presence of the indicated concentrations of 991. *A*: IB analysis was performed with 20 μg lysates using the indicated antibodies. Representative blots are shown; *n* = 3. Shorter (1 min) and longer (5 min) exposure indicate the exposure time with X-ray film. *B-*-*G*: AMPK complexes were immunoprecipitated from 50 μg lysates with the indicated antibodies. AMPK activity (in duplicate) was measured in vitro as described in materials and methods. Results are expressed as means ± SE; *n* = 3. *Significance of AICAR or C13 versus the respective control condition (0, 0.01, or 0.1 μM 991); #significance of 991 versus the respective compound (AICAR or C13) or vehicle. *P* < 0.05.

We tested if the effect of 991 [which binds to a site formed between the small lobe of the α-subunit kinase domain and the β-subunit carbohydrate-binding module (46)] on AMPK activity could be further increased when it was coincubated with another compound, AICAR or C13 [which binds to the CBS domain of the γ-subunit, mimicking the action of AMP (18, 24)] in mouse primary hepatocytes. Hepatocytes were incubated with increasing concentrations of 991 (ranging from 0 to 0.3 μM) in the presence or absence of AICAR (0.1 mM) or C13 (10 μM) for 1 h, and phosphorylation of AMPK and its bona fide substrates (i.e., ACC and RAPTOR) was assessed by immunoblot analysis as surrogate markers of cellular AMPK activity (Fig. 4*A*). Treatment of hepatocytes with 991 alone resulted in a slight increase in the phosphorylation of AMPK and RAPTOR only at 0.3 μM, whereas a robust increase in ACC phosphorylation was readily observed and saturated at a concentration of 0.03 μM 991. AICAR or C13 alone robustly increased T172 phosphorylation of AMPKα, and when 991 was coincubated, there was a modest additional dose-dependent increase in AMPKα phosphorylation (as judged by increased band density and slower-migrating species). Single AICAR or C13 treatment caused ACC phosphorylation to saturating levels. In contrast, RAPTOR phosphorylation was modestly increased by AICAR or C13 alone, and it was dose dependently increased when coincubations were carried out with 991 (Fig. 4*A*). These data are consistent with our previous observation that RAPTOR phosphorylation requires a higher threshold of AMPK activity than ACC phosphorylation in mouse hepatocytes (18).

To assess the effects of single or dual agonist treatment on the activities of specific AMPK isoforms/complexes in hepatocytes, we next performed an in vitro quantitative AMPK activity assay after immunoprecipitation with specific AMPK subunit isoform antibodies (Fig. 4, *B–G*). As anticipated, the activity of both AMPKα1- and AMPKα2-containing complexes (Fig. 4, *B* and *C*) resembled the results of AMPKα T172 phosphorylation (Fig. 4*A*) in response to compound treatments. Of note and consistent with our previous observation (18), incubation with C13 alone more robustly stimulated the activity of AMPKα1-containing complexes, whereas AICAR treatment increased the activation of AMPKα1 and AMPKα2 in a relatively similar manner. However, the absolute activity achieved by C13 was similar between α1 and α2, whereas AICAR caused much higher absolute activation for α2 (Fig. 4, *B* and *C*). In the mouse liver, AMPKβ1 is the predominant isoform, whereas expression of AMPKβ2 is marginal (9). Activity of AMPKβ1-containing complexes displayed a similar activation profile (Fig. 4*D*) to the activity of AMPKα1- and AMPKα2-containing complexes (Fig. 4, *A* and *B*). As expected, AMPK activity associated with AMPKβ2 complexes was much lower than AMPKβ1 complexes and was robustly activated by AICAR or C13. However, there was no further activation when 991 was incubated along with AICAR or C13 (Fig. 4*E*). This might be due to the fact that 991 has a higher affinity for AMPKβ1 (in cell-free assay) (46) and thus the low concentrations of 991 used were not sufficient to stimulate the activity of AMPKβ2-containing complexes. We then measured AMPK activity associated with AMPKγ1 or AMPKγ2 and observed that treatment with AICAR alone showed similar and significant increases in AMPK activity of complexes associated with both isoforms, although the extent of activation was higher in AMPKγ1-containing complexes (Fig. 4, *F* and *G*). C13 promoted a robust increase in the activity of the AMPKγ1-containing complex but not the AMPKγ2-containing complex. Moreover, robust increases (∼2- to 3-fold) in AMPK activity of AMPKγ1- or AMPKγ2-containing complexes were observed when 991 was combined in a dose-dependent manner (Fig. 4, *F* and *G*).

We observed that 991 dose dependently (0.01 and 0.1 μM) inhibited lipogenesis (34% and 63%, respectively), which was further reduced when it was coincubated with a low dose of AICAR (0.03 mM) or C13 (1 μM) (data not shown).

#### Enhanced activation of AMPKγ1- and AMPKγ3-containing complexes by dual compound treatment in C_2_C_12_ muscle cells.

**Fig. 5.**
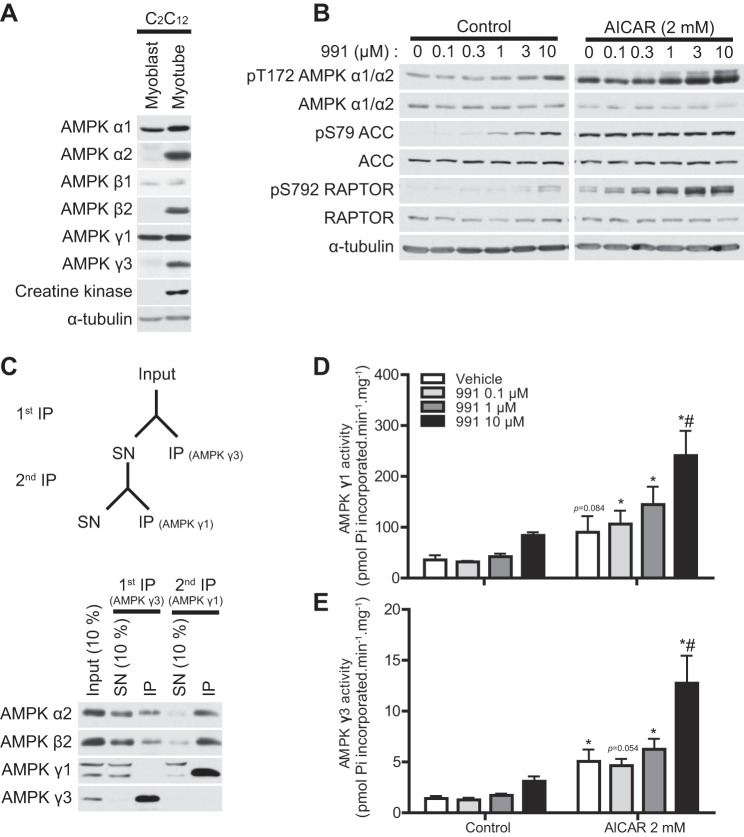
991 treatment enhances AMPK activity induced by AICAR in C_2_C_12_ muscle cells. *A*: IB analysis was performed with 20 μg lysates from C_2_C_12_ myoblasts or myotubes with the indicated antibodies. Representative blots are shown; *n* = 2 for each condition. Creatine kinase was used as a differentiation marker. *B*: IB analysis was performed with 20 μg lysates from C_2_C_12_ myotubes untreated (control) or treated with AICAR (2 mM) in the presence or absence of the indicated dose of 991 for 30 min. Representative blots are shown; *n* = 3 for each condition. *C*: schematic representation of the sequential immunoprecipitation protocol for the isolation of AMPKγ3 and AMPKγ1 from tissue lysates (*top*). AMPKγ3 and AMPKγ1 immune complexes were sequentially immunoprecipitated from 200 μg mouse gastrocnemius muscle lysate protein. IB analysis was then performed on the input (10% = 20 μg), the SN (10% = 20 μg), and the immunoprecipitates (IP) from the first and second immunoprecipitation using the indicated antibodies (*bottom*). *D*: AMPKγ3 and AMPKγ1 activity (in duplicate) from 200 μg C_2_C_12_ myotube lysate protein were measured after sequential immunoprecipitations as described in materials and methods. Results are expressed as means ± SE; *n* = 3. *Significance of AICAR versus the respective control condition (0 or 10 μM 991); #significance of 991 versus the respective control (0 or 2 mM AICAR). *P* < 0.05.

We next investigated the effects of dual compound treatment on AMPK activity in muscle cells. We first characterized the isoform-specific expression pattern of AMPK in C_2_C_12_ mouse myoblasts and myotubes. We observed that in myoblasts, expression of α1, β1, and γ1 was readily detectable and their levels did not alter when differentiated into myotubes. In contrast, expression of α2, β2, and γ3 was only detectable in myotubes but not in myoblasts (Fig. 5*A*). Consistent with the results obtained using mouse skeletal muscle tissue (Fig. 1*E*), levels of AMPKγ2 in C_2_C_12_ myoblasts and myotubes were undetectable (data not shown).

Incubation of C_2_C_12_ myotubes with 991 alone modestly increased AMPKα phosphorylation but only at a concentration of 10 μM (Fig. 5*B*). Treatment with 991 promoted dose-dependent increases in ACC and RAPTOR phosphorylation. Similar to the observations in hepatocytes (Fig. 4*A*) (18), in muscle cells, phosphorylation of RAPTOR also appeared to require a higher threshold of AMPK activity than ACC phosphorylation (Fig. 5*B*). Incubation with AICAR robustly promoted AMPKα phosphorylation, which was further increased in a dose-dependent manner in the presence of 991. AICAR (2 mM) alone readily saturated ACC phosphorylation, whereas RAPTOR phosphorylation was further increased in a dose-dependent manner in the presence of 991 (Fig. 5*B*). We then attempted to establish a sequential immunoprecipitation protocol to measure activities of AMPKγ1- and AMPKγ3-contaning complexes from small volumes of muscle cell/tissue extracts. As shown in Fig. 5*C* and consistent with our earlier observations (Fig. 2, *A* and *C*), incubation of lysates with AMPKγ3 antibody (in complex with protein G-Sepharose) selectively immunoprecipitated AMPKγ3-contaning complexes but not AMPKγ1-containing complexes. Using the supernatant after AMPKγ3 immunodepletion, AMPKγ1-containing complexes were efficiently immunoprecipitated (Fig. 5*C*). Activity of both AMPKγ1- and AMPKγ3-containing complexes tended to increase after treatment with 991 alone at a concentration of 10 μM (Fig. 5, *D* and *E*), consistent with the results of AMPKα phosphorylation (Fig. 5*B*). In contrast, 991 treatment only further enhanced the effect of AICAR on AMPK activity of AMPKγ1- and AMPKγ3-containing complexes at a concentration of 10 μM (Fig. 5, *D* and *E*).

#### Effect of dual compound treatment on AMPKγ1/γ3 activity and glucose transport in mouse skeletal muscle.

**Fig. 6.**
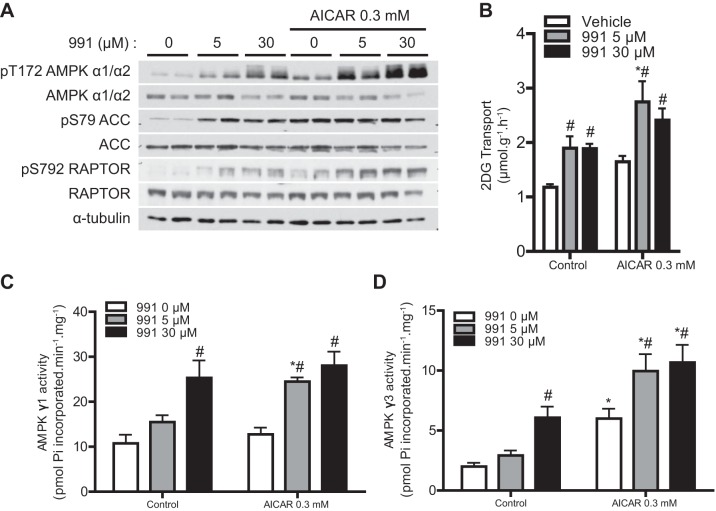
Dual compound treatment further increases AMPK activity and glucose transport in mouse skeletal muscle compared with single compound treatment. EDL muscles from C57BL/6 mice were isolated and incubated in the presence or absence of the indicated compounds (and concentrations) for 1 h followed by the 2-deoxyglucose (2-DG) transport assay as described in materials and methods. *A*: IB analysis was performed with 20 μg muscle lysate protein with the indicated antibodies. Representative blots are shown; *n* = 4. *B*: 2-DG transport (*n* = 4). *C* and *D*: AMPK complexes were immunoprecipitated from 200 μg muscle lysate protein using either AMPKγ1 (*C*) or AMPKγ3 (*D*) antibody. AMPK activity was measured as described in materials and methods. Results are expressed as means ± SE; *n* = 4. *Significance of AICAR versus the respective control condition (0, 5, or 30 μM 991); #significance between 991 and the respective vehicle condition (0 or 0.3 mM AICAR). *P* < 0.05.

We tested whether dual compound treatment further stimulated AMPK activity and glucose transport in mouse skeletal muscle. Isolated EDL muscle was incubated in the presence or absence of 991 with or without AICAR. 991 alone increased the phosphorylation of AMPKα and RAPTOR in a dose-dependent manner, whereas 5 μM 991 already increased phosphorylation of ACC at maximum levels. AICAR (0.3 mM) alone robustly increased the phosphorylation of AMPKα, ACC, and RAPTOR (Fig. 6*A*). Cotreatment of EDL with 991 and AICAR further promoted the phosphorylation of AMPKα and RAPTOR compared with single treatments. Incubation of EDL with 991 (5 and 30 μM) caused an almost twofold increase in 2-deoxyglucose transport, as previously described (22), although there was no dose-response effect under the condition we tested. AICAR also tended to increase 2-deoxyglucose transport ∼1.5-fold (not statistically significant), which was further enhanced (∼1.5-fold) by 991 (5 and 30 μM; Fig. 6*B*). AMPKγ1-containing activity was increased (∼2.5-fold) with 30 μM 991 (Fig. 6*C*). While AICAR (0.3 mM) alone showed no significant increase, when coincubated with a lower concentration of 991 (5 μM but not 30 μM), it significantly promoted AMPKγ1-containing activity compared with single 5 μM 991 treatment (Fig. 6*C*). 991 (30 μM) caused a significant increase in AMPKγ3-containing activity. AMPKγ3-containing activity was robustly increased by incubation with AICAR (∼4-fold) and was further increased in the presence of 991 (5 and 30 μM; Fig. 6*D*).

#### Additive effects of 991 treatment and contraction on AMPK activity and glucose transport in mouse skeletal muscle.

**Fig. 7.**
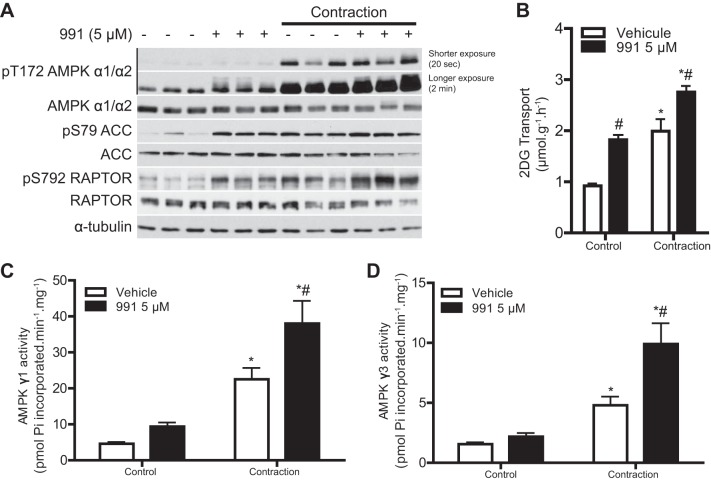
991 and contraction additively increase AMPK activity and glucose transport in mouse skeletal muscle. EDL muscles from C57BL/6 mice were isolated and incubated in the presence or absence of 991 (5 μM) for 45 min. Muscles were then either electrically stimulated to induce contraction or further incubated (no electrical stimulation) for 15 min. 2-DG transport was measured during the last 10 min of contraction or incubation as described in materials and methods. *A*: IB analysis was performed on 20 μg muscle lysate protein with the indicated antibodies. Representative blots are shown; *n* = 8. Shorter (20 s) and longer (2 min) exposure indicate the exposure time with X-ray film. *B*: 2-DG transport (*n* = 8). *C* and *D*: AMPK complexes were immunoprecipitated from 200 μg muscle lysate protein using either AMPKγ1 (*C*) or AMPKγ3 (*D*) antibody. AMPK activity was measured as described in materials and methods. Results are expressed as means ± SE. *Significance of contraction versus the respective control condition (0 or 5 μM 991); #significance of 991 versus the respective vehicle condition with or without 1-Hz contraction. *P* < 0.05.

We next examined if the effect of 991 on AMPK activity and glucose transport could be further stimulated when combined with contraction in mouse skeletal muscle ex vivo. We have previously observed that intense tetanic contraction [100 Hz, which causes a robust (∼4-fold) increase in muscle glucose uptake] failed to elicit an additive effect when combined with various doses of 991 (22). It might be the case that under such a high-intensity contraction protocol, glucose transport through GLUT4 translocation got saturated and thus there was no room for further increase when 991 was added. Therefore, we used a low/moderate-intensity twitch (1 Hz) contraction protocol, which causes submaximal glucose transport (42). Isolated mouse EDL muscle was incubated with 991 (5 μM) for 45 min, and the muscle was then either electrically stimulated (contraction) or further incubated (control) with or without 991 for 15 min. As shown in Fig. 7*A*, 991 treatment alone modestly increased AMPKα phosphorylation (longer exposure), which was associated with increases in the phosphorylation of ACC and RAPTOR. Contraction increased AMPK phosphorylation much more robustly compared with 991, whereas the increases in phosphorylation of ACC and RAPTOR were similar to those induced by 991. Treatment with 991 and electrical stimulation in combination resulted in modest additional increases in the phosphorylation of AMPKα and RAPTOR (Fig. 7*A*). Treatment with 991 or contraction alone significantly increased 2-deoxyglucose transport to the same extent (∼2-fold; Fig. 7*B*). When 991 treatment and contraction were combined, an additive increase in 2-deoxyglucose transport was observed.

We next assessed if the activities of AMPKγ1- and AMPKγ3-contaning complexes were increased in response to contraction and whether the increase was enhanced when combined with 991 treatment. 991 treatment resulted in a modest but nonsignificant increase in AMPK activity of AMPKγ1- and AMPKγ3-containing complexes (Fig. 7, *C* and *D*). Contraction robustly increased activities of both AMPKγ1- and AMPKγ3-containing complexes, which were further increased when 991 treatment and contraction were combined (Fig. 7, *C* and *D*).

## DISCUSSION

There has been keen interest in developing AMPK-activating drugs for therapeutic use in treating metabolic diseases such as type 2 diabetes. AMPK is involved in a plethora of biological/physiological events, which could partly be explained by the fact that AMPK exists theoretically as 12 distinct trimeric complexes (excluding splice variants) that are uniquely distributed across different cell types/tissues. Therefore, activators that specifically target different isoforms/complexes of AMPK hold promise in drug development for the treatment of specific symptoms/disorders, possibly with fewer off-target effects. Isoform-specific activators also represent valuable research tools to study AMPK functions in intact cells/tissues. Although several studies have reported expression profiles of AMPK isoforms/complexes in mouse skeletal muscles (43), the specificity of the antibodies used was not perfectly documented and validated. In the present study, we first carried out a comprehensive characterization/validation of AMPK isoform-specific antibodies for immunoblot analysis and immunoprecipitation using tissue extracts from AMPK isoform-specific KO mouse models as negative controls. Taking advantage of the validated antibodies, we also assessed the expression of AMPK isoforms in different mouse skeletal muscles and other tissues. It has been controversial whether the AMPK γ2-isoform is expressed in muscle. One study showed the presence/detection of AMPKγ2 in mouse skeletal muscle tissues assessed by immunoblot analysis but failed to detect it in AMPK complexes associated with α1/α2 or β1/β2 (43). Using a newly generated AMPKγ2-deficient mouse model, we showed that the full-length AMPKγ2 variant is not expressed at detectable levels in mouse hindlimb skeletal muscles. We speculate that the previously detected immunoreactivity was nonspecific. Using AMPKγ3 KO muscle lysates, we also confirmed that AMPKγ3 is only detectable in skeletal muscle and is expressed in glycolytic (e.g., EDL) and glycolytic-oxidative mixed (e.g., gastrocnemius) but not oxidative (e.g., soleus) mouse muscles, as previously described (2, 49). Moreover, we observed that AMPKγ3 (as well as AMPKα2 and AMPKβ2) is not detectable in C_2_C_12_ myoblasts but is induced upon differentiation into myotubes. Although the molecular mechanism by which AMPKγ3 expression is controlled remains unknown, the involvement of coactivator-associated arginine methyltransferase 1/protein arginine methyltransferase 4 has been shown in the regulation of AMPKγ3 expression and that of other genes involved in glycogen metabolism in C_2_C_12_ muscle cells (44).

We (8, 11, 41) and others (35) have recently shown that dual AMPK-activating compound treatment (A769662 and AICAR) resulted in additive/synergistic increases in cellular AMPK activity and downstream physiological effects such as the inhibition of lipogenesis in hepatocytes (8) and glucose transport in cardiomyocytes (41). One limitation of using A769662 in dual treatment is that it is only effective in cells where β1 is predominantly expressed. Although both A769662 and 991 bind to the ADaM site (4, 23, 46), 991 is much more potent than A769662 (in assays monitoring allosteric activation and protection against dephosphorylation using recombinant α2β1γ1 complexes) (46). However, it should be noted that even though 991 increases AMPK activity of both β1- and β2-containing complexes, it binds to β1-containing complexes ∼10 times stronger than to β2-containing complexes in cell-free assays (46) (which possibly explains its weaker activation of β2-complexes versus β1-complexes in vitro and also in intact cells; Fig. 4*E*). Nonetheless, we demonstrated that 991 increases AMPK activity of both β1- and β2-containing complexes and thus, as anticipated, it also activates AMPKα1-/α2- and AMPKγ1-/γ2-containing complexes in hepatocytes. We also showed that the effects of 991 on cellular AMPK activity in hepatocytes can be further enhanced (as judged by a dose-dependent increase in RAPTOR phosphorylation) in the presence of AICAR or C13. Moreover, we showed here that γ1- and γ3-complex activity as well as glucose transport were additively/synergistically enhanced when 991 and AICAR were combined, indicating that the dual treatment approach can work in various different cell systems irrespective of the composition of the β-subunit.

Recently, the effect of another small-molecule AMPK activator, PT-1, on AMPK activity and glucose uptake in mouse skeletal muscle was investigated (20). An unexpected observation reported in that study (20) was that although PT-1 was initially thought to activate AMPK by direct binding between the kinase and auto-inhibitory domains of the α-subunit (28), Jensen et al. showed that PT-1 failed to activate AMPK directly but rather indirectly activated it by inhibiting the respiratory chain and increasing cellular AMP-to-ATP and/or ADP-to-ATP ratios (20). Nevertheless, they showed that PT-1-activated AMPK associated only with AMPKγ1- and not AMPKγ3-containing complexes, which resulted in no significant increase in ACC2 and TBC1 domain family member 1 phosphorylation and no increase in glucose transport in mouse skeletal muscle. Although the mechanism by which PT-1 preferentially activates AMPKγ1-containing complexes (particularly in mouse skeletal muscle) is unknown, the study by Jensen et al. (20) indicated that activation of AMPKγ3-containing complexes is required to increase glucose transport in intact mouse skeletal muscle. It has been well documented that AICAR stimulates glucose transport in mouse skeletal muscle in an AMPK-dependent mechanism based on a study using AMPK-deficient genetic mouse models (30). It has recently been shown that 991 treatment stimulates glucose transport in mouse skeletal muscle and that the 991-mediated increase in glucose transport was absent in AMPKα1/α2 KO cultured myotubes (22). We showed in mouse EDL muscle that both 5 μM 991 and contraction significantly increased glucose transport to a similar extent (∼2-fold), although AMPK activity of γ1-and γ3-containing complexes was much greater in contracted muscles. However, it should be noted that 991 increases AMPK activity not only through protection against dephosphorylation leading to AMPKα T172 phosphorylation-dependent activation but also via potent allosteric stimulation through the ADaM site (46). Given that measurement of AMPK activity in vitro only assesses phosphorylation (T172)-dependent activation, it is possible that α2β2γ1/α2β2γ3 complexes are allosterically activated in intact muscle by 991. This may at least partly explain why phosphorylation of bona fide AMPK substrates (ACC and RAPTOR) and the extent of glucose transport were comparable between 991- and contraction-stimulated muscles. It would be of interest to identify γ1-and γ3-complex-specific substrate(s) (if any) and monitor their cellular activity upon 991 treatment through its/their phosphorylation by immunoblot analysis. In addition, whether AMPKγ3 is necessary for 991-stimulated glucose uptake, like AICAR, would be interesting to test using an AMPKγ3 KO mouse model.

In summary, we demonstrated that 991 treatment increases AMPK activity of both AMPKγ1- and AMPKγ3-containing complexes in mouse skeletal muscle. We also showed that dual treatment with 991 and AICAR or 991 and contraction augments AMPK activation and glucose transport compared with single treatment. The dual treatment approach has proven to be useful to robustly activate cellular AMPK, thereby facilitating the identification of new AMPK substrates in different cell types (7, 17). Next, it would be of interest to test if dual treatment (combinations of various compounds or compound plus exercise/contraction) also promotes AMPK activity and glucose transport in vivo.

## GRANTS

A. Yavari is supported by the United Kingdom (UK) National Institute for Health Research. A. Yavari (RE/08/004) and H. Ashrafian acknowledge support from the British Health Foundation Centre of Research Excellence (Oxford, UK). Y.-C. Lai was supported by the Fonds de la Recherche Scientifique (FNRS; Belgium) and Interuniversity Attraction Poles (IAP) Programme of the Belgian Science Policy (P7/13). S. Kviklyte was also supported by IAP programme P7/13. This work was funded by IAP Belgian Science Policy (P7/13), by the Directorate General Higher Education and Scientific Research French Community of Belgium, and by the FNRS (Belgium) under Grants 3.4518.11 and T.0008.15. T. E. Jensen and A. L. B. Madsen were supported by a Novo Nordisk Foundation Excellence project grant and the Danish Diabetes Academy. E. A. Richter was supported by a Grant 4183-00249B from the Danish Council for Independent Research/Medicine.

## DISCLOSURES

L. Bultot, C. Collodet, M. Deak, and K. Sakamoto are employees of the Nestlé Institute of Health Sciences SA (Switzerland).

## AUTHOR CONTRIBUTIONS

L.B., T.E.J., and K.S. conception and design of research; L.B., T.E.J., Y.-C.L., A.L.M., C.C., S.K., M.D., A.Y., M.F., S.G., and M.B. performed experiments; L.B., T.E.J., Y.-C.L., A.L.M., C.C., E.A.R., and K.S. analyzed data; L.B., T.E.J., Y.-C.L., C.C., A.Y., M.F., H.A., M.R., E.A.R., and K.S. interpreted results of experiments; L.B. and C.C. prepared figures; L.B. and K.S. drafted manuscript; L.B., T.E.J., Y.-C.L., M.D., A.Y., M.F., M.R., E.A.R., and K.S. edited and revised manuscript; L.B., T.E.J., Y.-C.L., A.L.M., C.C., S.K., M.D., A.Y., M.F., S.G., M.B., H.A., M.R., E.A.R., and K.S. approved final version of manuscript.
